# Redescription of *Stenothyra glabra* A. Adam, 1861 (Truncatelloidea, Stenothyridae), with the first complete mitochondrial genome in the family Stenothyridae

**DOI:** 10.3897/zookeys.991.51408

**Published:** 2020-11-11

**Authors:** Lu Qi, Lingfeng Kong, Qi Li

**Affiliations:** 1 Key Laboratory of Mariculture, Ministry of Education, Ocean University of China, Qingdao, 266003, China Ocean University of China Qingdao China; 2 Laboratory for Marine Fisheries Science and Food Production Processes, Qingdao National Laboratory for Marine Science and Technology, Qingdao, 266237, China Qingdao National Laboratory for Marine Science and Technology Qingdao China

**Keywords:** Micromollusks, mitogenome, phylogeny, systematics

## Abstract

In this study, *Stenothyra glabra* belonging to the truncatelloid family Stenothyridae is redescribed using morphological characters from the shell, operculum, and radula. The species is distinguished from other species in the group by its shell without spotted spiral lines and by its dome-shaped, mostly smooth, protoconch with some pits. Together with the morphological description, the complete mitogenome for the species is provided, which fill a knowledge gap in Stenothyridae. The mitogenome of *S. glabra* is 15,830 bp in length and has a circular structure. It contains 37 genes: 22 transfer RNA genes (tRNAs), two ribosomal RNA genes (rRNAs), and 13 protein-encoding genes (PCGs). The overall A+T content of the mitogenome is 68.9%. Molecular phylogenetic analysis and COI sequence divergence separate *S. glabra* from its congeners and show that *S. glabra* and S. cf. divalis form a sister clade.

## Introduction

The Stenothyridae are a family of small to minute-sized gastropods found in intertidal, shallow-water aquatic habitats and brackish estuaries in Asia and Australia (Golding 2014). A preliminary investigation through the World Register of Marine Species (WoRMS) recovered 212 species-level names, belonging to ten genus-level groups, including approximately 80 extant species from four genera, while a previous estimate of stenothyrid diversity recognized approximately 60 species globally (Strong et al. 2008). Almost all recent species are placed in the genus *Stenothyra* Benson, 1856, involving approximately 75 extant species. Due to the groups being small in size and exhibiting relatively simple morphologies, only a few stenothyrids have been described in detail (Kosuge 1969; Davis et al. 1986, 1988; Golding 2014).

*Stenothyra glabra* A. Adam, 1861 is a brackish-water species, which is thought to be the only *Stenothyra* species distributed along the coast of north China (Zhang et al. 1964; Qi et al.1989; Zhang et al. 2016). The brief original description (without illustration) by A. Adams (1861) is inadequate in that many features were not evaluated or included. On the other hand, stenothryid species are so similar in morphology that it is relatively difficult to distinguish them with the naked eye, so it is important to obtain clear illustrations and to redescribe the species in detail. Moreover, the species have relatively little molecular data available at present, and not much is known about phylogenetic relationships within the family. The aims of this study were thus: (a) to redescribe *S. glabra* based on specimens collected from the coast of north China using Scanning Electron Microscope images of the shell, protoconch, operculum, and radula; (b) to sequence the complete mitogenome of *S. glabra* and fill a knowledge gap; and (c) to use molecular data to reconstruct phylogenetic relationships and clarify the position of *S. glabra*.

## Materials and methods

### Taxon sampling and processing

Samples were collected from a mud flat in the Yellow River estuary (37°49.3676'N, 119°09.0351'E), Shandong, China on 17 Sept. 2017 and Ganyu (34°51.9126'N, 119°12.681'E), Jiangsu, China on 16 Sept. 2018. All specimens were preserved in 95% non-denatured ethanol and deposited in the Laboratory of Shellfish Genetics and Breeding (**LSGB**), Fisheries College, Ocean University of China, Qingdao, China. The following standard measurements were taken using a stereomicroscope with an eyepiece micrometer. The number inside the brackets indicates the number of specimens in each lot. Total genomic DNA was extracted from entire animals with the TIANamp Marine Animals DNA Kit (Tiangen Biotech, Beijing, China) according to manufacturer’s protocol, and stored at -4 °C for short-term use. The Scanning Electron Microscope (SEM) was used to examine shells, radulae, and opercula based on the methods given by Geiger et al. (2007) and Geiger (2012). Briefly, for SEM studies of radula, the tissue surrounding the radula was dissolved by proteinase K when extracting DNA from entire animals using the TIANamp Marine Animals DNA Kit. The radula was precipitated to the bottom of the centrifugal tube after centrifuge separating, and was collected using a pipette. Then the radula was washed in drops of water or 10% KOH on a glass histology slide. Shells, radulae, and opercula were mounted on stubs, thinly coated with gold, and examined using a TESCAN VEGA3 SEM.

### Sequencing, assembly, and annotation

Library construction and sequencing were performed by Beijing Novogene Technology Co., Ltd (China) from total genomic DNA on the HiSeq X platform (Illumina Inc.) with 150-bp paired-end reads. Raw data were initially quality-trimmed using Trimmomatic v0.36 (Bolger et al. 2014). Resulting clean reads were assembled using the software SPAdes 3.13.0 (Bankevich et al. 2012) with default settings. The complete mitochondrial genome was identified using BLASTN (Altschul et al. 1997) and the previously published mitochondrial genome of *Oncomelania hupensis robertsoni* (EU079378.1) was used as the reference. The mitogenomes were annotated using MITOS WebServer (http://mitos.bioinf.uni-leipzig.de/index.py) (Bernt et al. 2013) to identify protein-coding genes (PCGs), ribosomal RNA (rRNAs), and transfer RNA (tRNAs) genes. Gene limits were refined by comparison with orthologous mtDNA sequences of closely related species of Truncatelloidea and using BLASTX (Altschul et al. 1997) against the non-redundant protein sequences database in GenBank. Two ribosomal RNA genes (rrnL and rrnS) were identified by alignment with published Truncatelloidea mitogenomes, and their ends were assumed to extend to the boundaries of their flanking genes. The tRNAs were also annotated with ARWEN v1.2 (Laslett and Canbäck 2008) and tRNAscan-SE v1.21 (Lowe and Eddy 1997) and manually curated when inconsistencies were detected between tools. Base composition and codon usage were analyzed with MEGA 6.0 (Tamura et al. 2013). The GC and AT skews were calculated using the formulae: AT skew = (A-T)/(A+T) and GC skew = (G-C)/(G+C) (Perna and Kocher 1995). The circular map of the *S. glabra* mitogenome was drawn with the mitochondrial visualization tool CGView (Stothard and Wishart 2005; http://stothard.afns.ualberta.ca/cgview_server/). In addition, contigs of 28S rRNA genes were identified using BLASTN with sequences from Golding (2014) serving as the reference against the assembled genomic data, followed by manual annotation of gene boundaries.

### Phylogenetic analysis

No mitochondrial genomes of stenothyrids were available from GenBank, so we reconstructed the phylogenetic trees of the genus *Stenothyra* using COI, 16S, and 28S fragments, combining our DNA sequences with sequences from GenBank that included eleven stenothyrid taxa and one anabathrid species, *Pisinna punctulum*, as the outgroup (Table 1). Alignment of all stenothyrid and outgroup sequences was performed using default parameters in MEGA 6.0 and proofread by eye. Aligned COI sequences were translated using the invertebrate mitochondrial code (NCBI translation code 5) to ensure stop codons or frameshift mutations were not present.

**Table 1.** GenBank accession numbers for specimens included in the molecular analyses. For COI and 16S, see GenBank accession number of the mitochondrial genome (MN548735).

Family	Species	COI	16S	28S
Stenothyridae	*Stenothyra glabra*	–	–	MT090057
	*Stenothyra australis*	KC439692	KC439814	KC439915
	*S. gelasinosa gelasinosa*	KC439704	KC439826	KC439917
	*S. gelasinosa phrixa*	KC439717	KC439836	KC439920
	*S. gelasinosa apiosa*	KC439720	KC439842	KC439921
	*S. paludicola topendensis*	KC439731	KC439853	KC439922
	*S. paludicola timorensis*	KC439733	KC439855	KC439923
	*Stenothyra monilifera*	KC439735	KC439857	KC439924
	Stenothyra cf. polita	KC439737	KC439859	KC439926
	*Stenothyra* sp. ‘* johor*’	KC439740	KC439862	KC439927
	Stenothyra cf. glabra	KC439741	KC439863	KC439928
	Stenothyra cf. divalis	KC439744	KC439866	KC439929
	*Pisinna punctulum*	KC439794	KC109968	KC110020

The best partition schemes and best-fit models of substitution for the data sets for phylogenetic analyses were identified using Partition Finder 2 (Lanfear et al. 2017) according to the Bayesian Information Criterion (BIC; Schwarz 1978). For the data sets analyzed at nucleotide levels, all genes were separated in the partitions (16S, 28S, COI). In addition, For the COI gene, these three partition schemes at nucleotide level were tested considering first, second and third codon positions separately.

Phylogenetic analyses were carried out using maximum likelihood (ML) and Bayesian Inference (BI) methods. ML analyses were performed with IQ-TREE (Nguyen et al. 2014) using the partition schemes and model (Table 2), and with 1000 Ultrafast bootstraps. The BI tree reconstruction was performed in MrBayes v3.2 (Ronquist and Huelsenbeck 2003) with two runs, each with four Markov Chain. All partitions were allowed to have their own set of parameters and to evolve under different rates. The analysis was run for ten million generations, sampling trees every 1000 generations. The initial 25% of the trees were discarded as burn-in and the remaining trees were used to generate a 50% majority rule consensus tree with nodal confidence assessed with posterior probabilities (BPP). Bayesian runs achieved sufficient convergence by ascertaining that the average standard deviation of split frequencies between chains was below 0.01 at the end of the runs and that the potential scale reduction factor of each parameter was 1.00. Trees were visualized using FigTree v1.3.1 and rooted using the outgroup species. Because these sequences are short and derived from closely related, so the *p*-distances are used as a simple measure of pairwise sequence divergences (Srivathsan and Meier 2012).

**Table 2.** The best partition schemes and best-fit models of substitution for the data sets.

Data set	Set Partition	Best Model
Best Partition to rRNA genes	16s	GTR+I+G
	28s	GTR+I+G
Best Partition to COI gene at nucleotide level	cox1 1^th^	GTR+I
	cox1 2^th^	F81
	cox1 3^th^	HKY+G

## Results

### Systematics

**Stenothyridae Tryon, 1866**

#### Stenothyra

Taxon classificationAnimaliaLittorinimorphaStenothyridae

Benson, 1856

AC0E1156-3939-5701-BA92-D1E5313C6FBB

##### Type species.

*Stenothyra delate* (Benson, 1837) from the delta of the Ganges (Benson 1837).

#### Stenothyra glabra

Taxon classificationAnimaliaLittorinimorphaStenothyridae

A. Adams, 1861

FEDD419D-DAF5-5F5E-99A7-01C26E189F94

Figures 1 , 2 , 3

Stenothyra glabra A. Adam, 1861: 307; Yen 1939: 45, pl. 4, fig. 15; Yen 1942: 197, pl. 14, fig. 44; Zhang et al. 1964: 61; Qi et al. 1989: 32–33, fig. 30; Zhang et al. 2016: 60–61.

##### Material examined.

China • 4, specimens; Shandong province, Dongying, Yellow River estuary mud flat; 37°49.367'N, 119°09.035'E; 17 Sept. 2017; Lu Qi leg.; LSGB S1702; • 6, specimens; Jiangsu province, Ganyu beach; 34°51.912'N, 119°12.681'E; 16 Sept. 2018; LSGB G1801.

##### Original description (verbatim).

“S. testa oblonga, laevi, polita, semipellucida, aurantiaca; anfractibus 4½, convexis, supremis transversim obsolete striates; suturis marginatis; peritremate continuo; anfraetu ultimo ad aperturam concentrice striato” (A. Adams 1861).

##### Diagnosis.

Shell ovate, dorso-ventrally compressed, with well-inflated body whorl and narrowly constricted aperture, without dotted spiral lines. Dome-shaped, smooth protoconch (1¾ whorls) with some pits. Posterior foot pointed, with metapodial tentacle.

##### Description.

**Figure 1.**
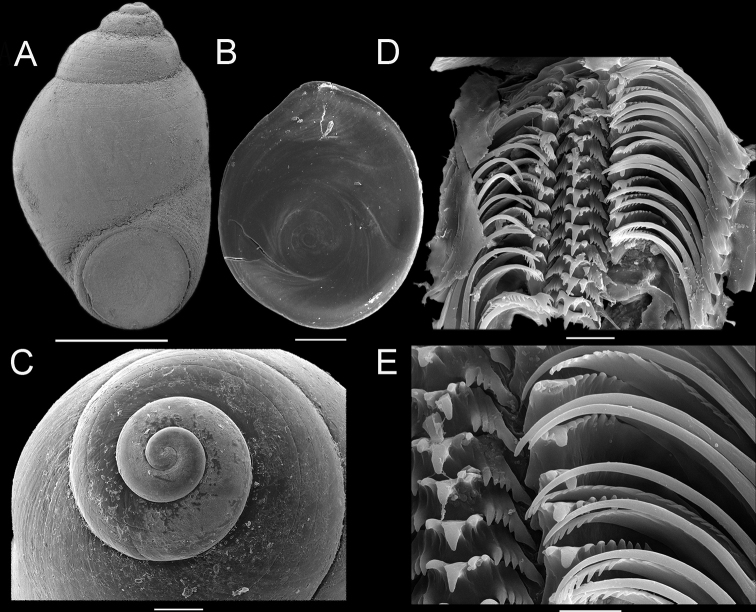
*Stenothyra glabra* A. Adams, 1861 **A** shell LSGB-G1801-4 **B** exterior surface of operculum **C** protoconch **D, E** radula. Scale bars: 1 mm (**A**); 200 μm (**B**), 200 μm (**C**), 20 μm (**D**), 10 μm (**E**).

***Shell*** minute (2.89±0.14 mm in height; 1.75±0.07 mm in width), ovate-conic, rather thick, dorso-ventrally compressed, with rounded to angled inflation of last whorl; up to five whorls including protoconch, convex whorls, sutures moderately deep; Surface smooth, yellowish brown, sculpture not dotted lines but continuous spiral grooves (Fig. 1A). The aperture abruptly descending, contracted, and nearly circular; peristome continuous, showing a weak triangular area; outer lip with marked grooves (Fig. 1A).

***Operculum*** ovate, yellowish, translucence, with very weak angulation aligning with posterior apex of aperture; nucleus of the exterior surface is close to the inner lip, paucispiral (Fig. 1B).

***Protoconch*** dome-shaped; smooth, 1¾ to 2 whorls; Small pits apparently exist in a small central part of protoconch (Fig. 1C).

***Radula*.** Radular teeth interlocked moderately in unfolded condition (Fig. 1D). Central tooth 1-2+1+1-2 (Fig. 1D, E); cusp with central denticle largest, 1–2 smaller ones on each side, basal denticles diminishing outwardly. Lateral teeth 2-3+1+6-8, apical ones largest, 2–3 denticles along inner edge of cusp, 6–8 along outer edge. Marginal teeth without groove; inner marginal teeth with ~20 cusps on tip and distal half of outer edge; outer marginal teeth with ~10 cusps on distal third of inner edge.

##### Type locality.

Estuary of the Pei-ho River (also known as the Hai River in the current name), North China.

##### Geographic distribution.

From Fujian to Hebei on coast of China (A. Adams 1861; Yen 1939; Qi et al.1989; Yuan et al. 2002; Bao et al. 2007); Japan (Kuroda 1962).

##### Ecology.

Inhabiting on the surface of mud flat or attaching to the under-surface of floating leaves in the freshwater estuary.

##### Remarks.

The type locality of *Stenothyra glabra* A. Adams, 1861 is “estuary of the Pei-ho, North China”, which is on the coast of the Bohai Sea. One of the localities in this study, Yellow River estuary, is adjacent to the type locality. Moreover, the shells are very similar in size, shape, and microsculpture when compare with the descriptions (A. Adams 1861; Yen 1939; Yen 1942; Zhang et al. 1964), as well as with the figure of A. Adam’s type (Yen 1942: 197, pl. 14, fig. 44). We believe that specimens collected in this study belong to a common species along the coast of the Yellow and Bohai seas in China, and is conspecific with the type material.

The radular morphology is one of the diagnostic morphological characters, but the Rachidian tooth and general radular shape of *S. glabra* appear similar to that of other *Stenothyra* species. This may be due to similarities in habit, substrate, and diet, suggesting that species delimitation in micro-caenogastropods should not rely solely on radular morphology. In fact, recent work has shown that some microgastropods exhibit morphological stasis in response to environmental stability (e.g., Weigand et al. 2011). However, there are sufficient morphological grounds for separating this species, by the shell not having dotted spiral lines and by the dome-shaped, smooth protochonch bearing some pits.

##### Sequence divergence.

The pairwise distance between species or non-conspecific subspecies ranged from 9.1% (*Stenothyra glabra* vs. S. cf. divalis) to 16.1% (*S. gelasinosa apiosa* vs. *S. monilifera*). COI sequence divergence between conspecific subspecies ranged from 3.0% (*Stenothyra paludicola timorensis* vs. *S. paludicola topendensis*) to 5.7% (*Stenothyra gelasinosa apiosa* vs. *S. gelasinosa gelasinosa*) (Table 3). Comparing the sequence divergences of within-taxon and between-taxon provided a sound basis for determining specific and subspecific-level differences. 3%-6% was evidence of subspecific diversity and > 9% was found between species. In this study, the divergence between *S. glabra* and other species fell into the latter category, having a lowest divergence of 9.1%. Notably, the divergence between *Stenothyra glabra* and S. cf. glabra (KC439741) is 13.2%. Stenothyra cf. glabra was collected from Mai Po, Hong Kong, China (Golding 2014), and is likely a misidentified animal.

**Table 3.** Pairwise *p*-distance among species of *Stenothyra*.

	***S. australis***	**S. cf. divalis**	**S. cf. glabra**	**S. cf. polita**	***S. gelasinosa apiosa***	***S. gelasinosa gelasinosa***	***S. gelasinosa phrixa***	***S. glabra***	***S. monilifera***	***S. paludicola timorensis***	***S. paludicola topendensis***
*S. australis*											
S. cf. divalis	0.109										
S. cf. glabra	0.135	0.129									
S. cf. polita	0.135	0.138	0.152								
*S. gelasinosa apiosa*	0.126	0.132	0.141	0.149							
*S. gelasinosa gelasinosa*	0.105	0.109	0.126	0.146	0.057						
*S. gelasinosa phrixa*	0.121	0.120	0.141	0.155	0.049	0.052					
*S. glabra*	0.118	0.091	0.132	0.146	0.111	0.097	0.109				
*S. monilifera*	0.126	0.121	0.123	0.151	0.161	0.136	0.148	0.123			
*S. paludicola timorensis*	0.132	0.106	0.108	0.141	0.133	0.120	0.138	0.109	0.124		
*S. paludicola topendensis*	0.135	0.114	0.103	0.139	0.135	0.126	0.141	0.109	0.133	0.030	
*Stenothyra* sp. ‘* johor*’	0.117	0.118	0.118	0.129	0.136	0.115	0.126	0.115	0.112	0.120	0.114

## Mitogenome architecture

### Genome organization and base composition

**Figure 2.**
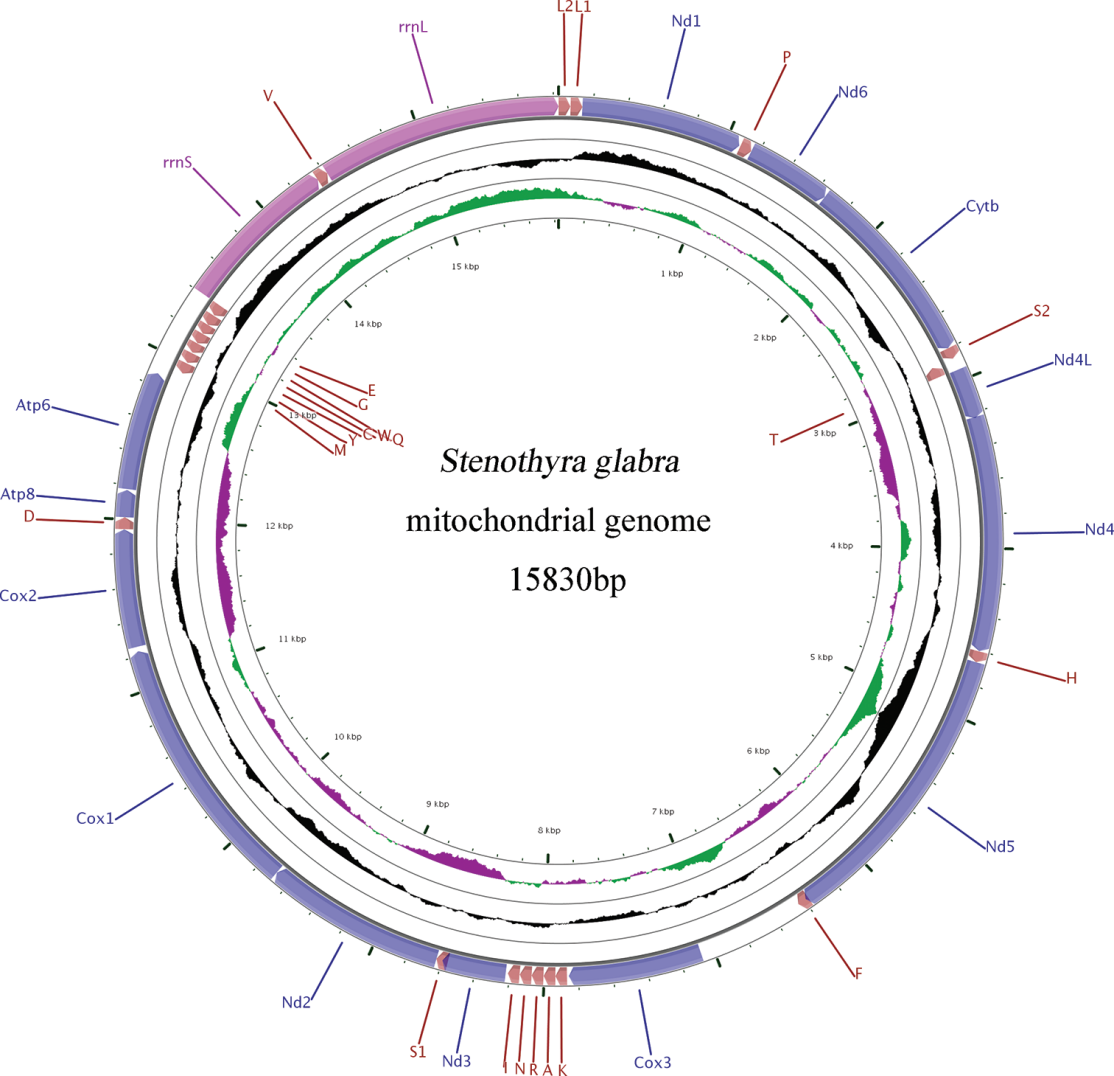
Map of the complete mitochondrial genome of *Stenothyra glabra*.

The circular mitogenome of *Stenothyra glabra* is 15,830 bp in size (GenBank accession number MN548735) and comprises 37 genes including 13 PCGs, 2 rRNAs genes, 22 tRNAs genes, and a putative control region (CR), typical of Gastropoda mitogenomes (Fig. 2). The CR is 633 bp and flanked by trnF and cox3.

### Protein-coding genes and codon usage

The total length of the concatenated 13 PCGs is 11271, with the average A+T content of 68.9%. ATG (for 12 PCGs) is the most commonly used start codon, whereas nad3 used TTG. The most frequent terminal codons are TAA (for 11 PCGs), whereas nad6 used a truncated T, nad4L used TAG, respectively (Table 4).

Codon usage, relative synonymous codon usage (RSCU), and codon family proportion (corresponding to the amino acids usage) of *S. glabra* is presented (Suppl. material 1). Serine (13.68%), phenylalanine (11.31%), leucine (11.15%) are the most frequent amino acids in the PCGs of *S. glabra*, whereas histidine (1.04%), glutamine (1.12%), arginine (1.12%) are relatively scarce.

**Table 4.** Annotated mitochondrial genome of *Stenothyra glabra*.

Gene	Direction	Position		Size	Intergenic	Condon		Anti-codon
		From	To		Nucleotides	Start	Stop	
trnL2	F	1	68	68	–	–	–	TAA
trnL1	F	70	138	69	1	–	–	TAG
nad1	F	139	1080	942	0	ATG	TAA	–
trnP	F	1088	1156	69	7	–	–	TGG
nad6	F	1158	1659	502	1	ATG	T	–
cytb	F	1660	2799	1140	0	ATG	TAA	–
trnS2	F	2800	2865	66	0	–	–	TGA
trnT	R	2866	2932	67	0	–	–	TGT
nad4L	F	2937	3234	297	4	ATG	TAG	–
nad4	F	3228	4601	1374	-5	ATG	TAA	–
trnH	F	4603	4667	65	1	–	–	GTG
nad5	F	4668	6392	1725	0	ATG	TAA	–
trnF	F	6376	6443	68	-15	–	–	GAA
cox3	F	7077	7856	780	633	ATG	TAA	–
trnK	F	7868	7934	67	11	–	–	TTT
trnA	F	7935	8002	68	0	–	–	TGC
trnR	F	8004	8072	69	1	–	–	TCG
trnN	F	8073	8141	69	0	–	–	GTT
trnI	F	8143	8210	68	1	–	–	GAT
nad3	F	8224	8597	374	13	TTG	TAA	
trnS1	F	8566	8633	68	-30	–	–	GCT
nad2	F	8634	9692	1059	0	ATG	TAA	–
cox1	F	9694	11229	1536	1	ATG	TAA	–
cox2	F	11256	11942	687	26	ATG	TAA	–
trnD	F	11944	12012	69	1	–	–	GTC
atp8	F	12013	12171	159	0	ATG	TAA	–
atp6	F	12177	12872	696	5	ATG	TAA	–
trnM	R	12930	12996	67	57	–	–	CAT
trnY	R	13002	13066	65	5	–	–	GTA
trnC	R	13071	13134	64	4	–	–	GCA
trnW	R	13136	13201	66	1	–	–	TCA
trnQ	R	13203	13264	62	1	–	–	TTG
trnG	R	13265	13331	67	0	–	–	TCC
trnE	R	13335	13403	69	3	–	–	TTC
rrnS	F	13404	14349	873	0	–	–	–
trnV	F	14349	14415	37	-1	–	–	TAC
rrnL	F	14416	15830	1415	0			

### Transfer and ribosomal RNA genes

The sizes of 22 tRNA genes of *S. glabra* range from 37 bp to 69 bp, comprising 1447 bp (9.1%) of the total mitogenome (Table 5). All 22 tRNA genes were identified and the secondary structures were shown in Suppl. material 2.

The genes rrnL and rrnS are 1415 bp and 946 bp in size, with 72.6% and 70.3% A+T content, respectively (Table 5). The location of rrnS is between trnE and trnV, and rrnL is located between trnV and trnL2 (Table 4); this is the same arrangement reported for Littorinimorpha (Osca et al. 2015).

**Table 5.** The nucleotide composition of *Stenothyra glabra* mitogenome.

Genes or regions	Size	Nucleotides composition (%)				A+T	AT Skew	GC Skew
		T	C	A	G	(%)		
Complete mitogenome	15830	41	12.5	28.7	17.8	69.7	-0.236	0.175
PCGs	11271	43	13	25.9	18.2	68.9	-0.248	0.167
tRNA genes	1447	33.7	13.5	34.7	18.1	68.4	0.014	0.144
rRNA genes	2361	35.6	10.9	36.1	17.4	71.7	0.0065	0.229
lrRNA	1415	36.7	10.7	35.9	16.7	72.6	0.0107	0.216
SrRNA	946	33.9	11.3	36.4	18.4	70.3	0.035	0.238
A+T-rich region	633	37.4	10	36.2	16.4	73.6	-0.017	0.246

### Phylogenetic analysis

**Figure 3.**
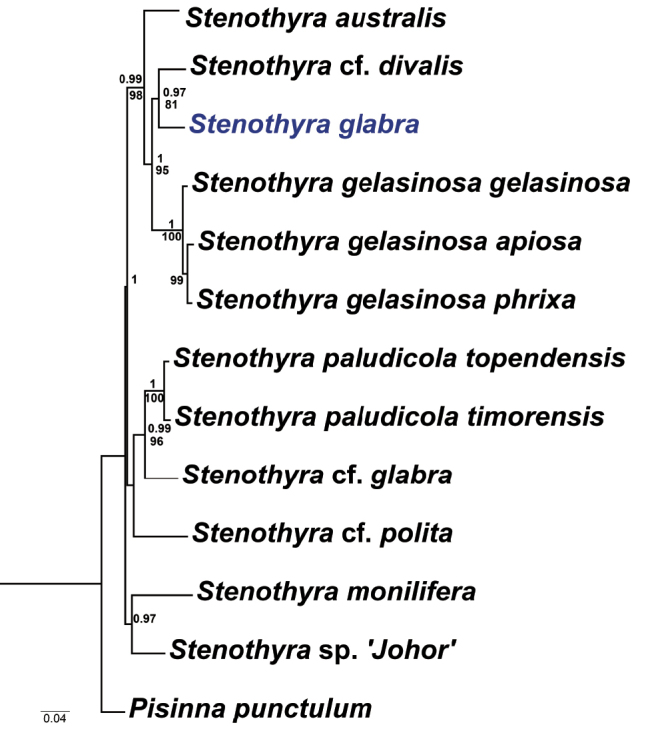
Summary tree from Maximum Likelihood analysis of concatenated COI, 16S and 28S sequences. Support indices are BI posterior probabilities (above nodes, > 0.9) and ML bootstraps (below nodes, > 70).

Phylogenetic reconstruction by BI and ML methods recovered mostly consensus trees with identical topologies, with the exception of one clade composed of *Stenothyra monilifera* and *S.* sp. ’* johor*’. Only the ML summary tree is shown here, labelled with both Bayesian posterior probabilities (BPP) and bootstrap support values (BS) generated by ML analysis (Fig. 3).

The phylogenetic analysis of stenothyrids, including most *Stenothyra* species with COI, 16S and 28S data in the NCBI, inferred the phylogenetic placement of *S. glabra*, and phylogenetic relationships of stenothyrids. *Stenothyra glabra* was recovered as the sister taxon to S. cf. divalis, and the COI divergence between them was 9.1%, the smallest value among those between *S. glabra* and other taxa of *Stenothyra* (Table 3). In the phylogeny, all *Stenothyra* taxa were split into three major clades. The basal clade included *S. monilifera* and *S.* sp. ’* johor*’ with relatively strong support in the BI analysis (BPP = 0.97), but with weak support in the ML analyses (BS < 70). *Stenothyra australis*, *S. gelasinosa*, S. cf. divalis, and *S. glabra* formed a well-supported clade (BPP = 0.99; BS = 98), while the third clade was composed of *S. paludicola topendensis*, *S. paludicola timorensis*, S. cf. glabra, and S. cf. polita, with a high support by BPP (> 0.98) and ML bootstrap values (> 90), except for the branches of Stenothyra cf. polita (BPP < 0.90, BS < 70). Our results are almost congruent with those acquired in the previous study (Golding 2014).

## Conclusion

The redescription of *Stenothyra glabra* based on SEM examination shows more morphological details of the shell, protoconch, and operculum. Radulae are described and illustrated herein for the first time. Additionally, the first mitochondrial genome of Stenothyridae will provide reference data for subsequent phylogenetic studies.

## Supplementary Material

XML Treatment for Stenothyra

XML Treatment for Stenothyra glabra

## Supplementary materials

Supplementary material 1 Relative synonymous codon usage (RSCU) of each amino acid in the mitogenome of *S. glabra* Data type image https://binary.pensoft.net/file/473851This dataset is made available under the Open Database License (http://opendatacommons.org/licenses/odbl/1.0/). The Open Database License (ODbL) is a license agreement intended to allow users to freely share, modify, and use this Dataset while maintaining this same freedom for others, provided that the original source and author(s) are credited.Lu Qi, Lingfeng Kong, Qi Li

Supplementary material 2 Secondary structure of tRNA in *S. glabra* mitogenome Data type doxc https://binary.pensoft.net/file/473852This dataset is made available under the Open Database License (http://opendatacommons.org/licenses/odbl/1.0/). The Open Database License (ODbL) is a license agreement intended to allow users to freely share, modify, and use this Dataset while maintaining this same freedom for others, provided that the original source and author(s) are credited.Lu Qi, Lingfeng Kong, Qi Li
