# Supplementary figures and images for: Redescription of Stenothyra glabra A. Adam, 1861 (Truncatelloidea, Stenothyridae), with the first complete mitochondrial genome in the family Stenothyridae
Source: Zookeys. 2020 Nov 11;991:69–83. doi: 10.3897/zookeys.991.51408 (PMC7674392; doi:10.3897/zookeys.991.51408)

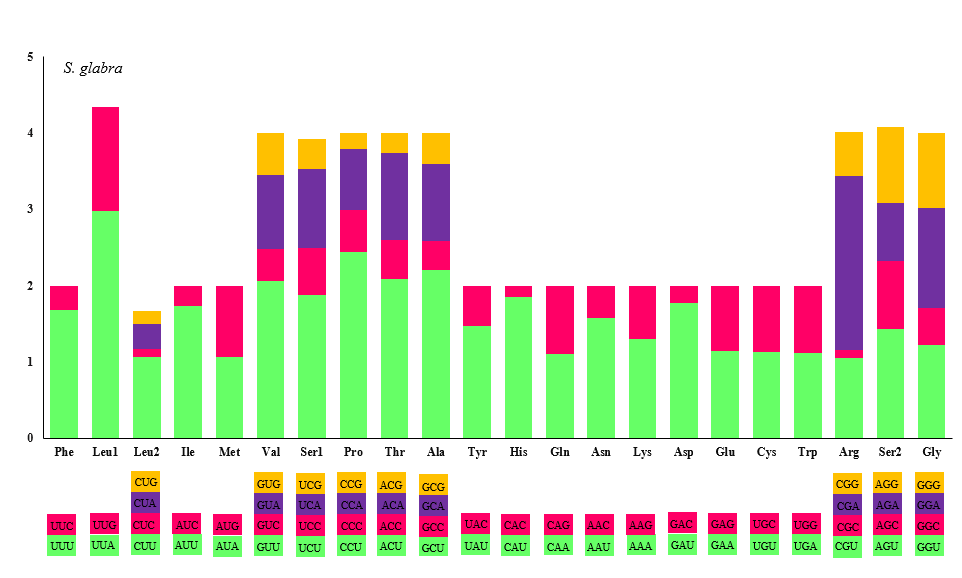

Supplement: Supplementary material 1 — Relative synonymous codon usage (RSCU) of each amino acid in the mitogenome of S. glabra [file zookeys-991-069-s001.png]
