# Supplementary material for: Redescription of Stenothyra glabra A. Adam, 1861 (Truncatelloidea, Stenothyridae), with the first complete mitochondrial genome in the family Stenothyridae
Source: Zookeys. 2020 Nov 11;991:69–83. doi: 10.3897/zookeys.991.51408 (PMC7674392; doi:10.3897/zookeys.991.51408)
Supplement: Supplementary material 2 — Secondary structure of tRNA in S. glabra mitogenome [file zookeys-991-069-s002.docx]

**Suppl. material 2**

Secondary structure of tRNA in *S. glabra* mitogenomes

1.

a

t-a

a-t

a-t

a-t

g-c

a-t

a-t

t-a tt

t tgatt a

a a !!!!! g

a attg actaa t

c !!!! t tt

t taac t

a c a

t-aa

t-a

a-t

g-c

a-t

t g

t a

gtc

mtRNA-Asp(gtc)

71 bases, %GC = 19.7

Sequence [2250,2320]

2.

a

t-a

t-a

g-c

t-a

a-t

a-t

a-t

a-t a

t ctct

aa a !!!! t

a tcga gaga t

t !!!! t a

t agct a

aaa g a

t-aa

t-a

g-c

g-c

g-c

t c

t a

cat

mtRNA-Met(cat)

69 bases, %GC = 27.5

Sequence c[3236,3304]

3.

a

t-a

g-c

t-a

t-a

a c

g-c

g-c t

t ctc a

a g !!! a

g gccg gag a

a :!!! g tt

c aggc c

c a a

g-ccc

t-a

a-t

g-c

a-t

c a

t g

gta

mtRNA-Tyr(gta)

65 bases, %GC = 50.8

Sequence c[3309,3373]

4.

t

a-t

c-g

c a

c-g

a-t

c-g

t-a

a-t a

t cttta

a a !!!!! t

t tttg gaaat

c !!!+ t t

aaat a

a a g

t-aa

c-g

g-c

a-t

a-t

g a

t a

gca

mtRNA-Cys(gca)

66 bases, %GC = 28.8

Sequence c[3377,3442]

5.

g

a-t

a-t

g-c

g-c

t-a

t-a

t-a t

t tttt t

a a !!!! a

a ttga aaaa t

a !!!! t ta

a aact a

a g a

c-ga

a-t

a-t

g-c

c-g

c a

t a

tca

mtRNA-(Stop|Trp)(tca)

66 bases, %GC = 24.2

Sequence c[3443,3508]

Overlap with 7: mtRNA-Gln(ttg) c[3506,3571]

6.

g

t-a

t-a

a-t

c-g

a a

a-t

g-c ac

c ctcc g

c g !!!! t

a tgtg gagg t

g +!!! a at

gcac g

a g a

a-ta

a-t

g-c

a-t

a-t

t t

t a

ttg

mtRNA-Gln(ttg)

66 bases, %GC = 40.9

Sequence c[3506,3571]

Overlap with 6: mtRNA-(Stop|Trp)(tca) c[3443,3508]

7.

a

a-t

t-a

c-g

t-a

c-g

t-a

t-a a

g aattt

cg a !!!!! a

t tatg ttaaa a

a +!!! a t

a gtac g

a a a

t-aa

t-a

t-a

g-c

c-g

c a

t a

tcc

mtRNA-Gly(tcc)

67 bases, %GC = 26.9

Sequence c[3572,3638]

8.

a

g+t

t-a

t-a

t-a

c-g

t-a

a-t t

t tattt c

at g !!!!! a

c tgtg ataaa a

t +!!! a tt

a gcac g

t a g

t-aa

t-a

a-t

g-c

g-c

t t

t a

ttc

mtRNA-Glu(ttc)

69 bases, %GC = 26.1

Sequence c[3642,3710]

9.

a

t-a

a-t

a-t

a-t

a-t

c-g

a-t a

t tgaat

aa a !!!!! t

a tacg actta g

t !!!! t g

t atgc a

a a a

t-aa

t-a

t-a

c-g

a-t

t c

t a

tac

mtRNA-Val(tac)

67 bases, %GC = 20.9

Sequence [4656,4722]

10.

t

t-a

a-t

t-a

a-t

g+t

a-t

g-c

a-t tg

t tttct a

ag g !!!!! a

a acg aaaga t

a !!! t tt

a tgc a

g a c

t-aa

t-a

a-t

g-c

g-c

t c

t g

taa

mtRNA-Leu(taa)

70 bases, %GC = 28.6

Sequence [6137,6206]

Overlap with 13: mtRNA-Leu(tag) [6206,6276]

11.

a

t-a

a-t

t-a

a-t

a-t

a-t

g-c

g+t ta

t tttct a

ag g !!!!! a

a acg aaaga a

t !!! t tt

a tgc a

ta a t

t-aa

t-a

a-t

g-c

g-c

t a

t g

tag

mtRNA-Leu(tag)

71 bases, %GC = 23.9

Sequence [6206,6276]

Overlap with 12: mtRNA-Leu(taa) [6137,6206]

12.

a

t-a

c-g

a-t

g+t

g-c

a-t

t-a

gt c tga

t accc t

a a !!!! a

a tttg tggg a

t !!!+ t ta

a aaat a

a a g

t-ac

t-a

a-t

g-c

c-g

a a

t a

tgg

mtRNA-Pro(tgg)

71 bases, %GC = 32.4

Sequence [7224,7294]

13.

a

a-t

g+t

g-c

c-g

t-a

a-t

t-a tt

t attcac a

g c !!!!! a

g tgcc aaagtg c

g +! ! t tt

gcag t

g c

t-a

c-g

t-a

g-c

t-a

c a

t a

tga

mtRNA-Ser(tga)

68 bases, %GC = 41.2

Sequence [8936,9003]

Overlap with 16: mtRNA-Thr(tgt) c[9003,9069]

14.

t

g-c

c-g

a-t

a-t

t-a

a-t

a-t t

t cttta

aa a !!!!! a

a ttcg gaaat t

a !!!! t t

t aagc a

a a g

t-ac

t-a

g-c

g+t

t+g

c a

t a

tgt

mtRNA-Thr(tgt)

67 bases, %GC = 26.9

Sequence c[9003,9069]

Overlap with 15: mtRNA-Ser(tga) [8936,9003]

15.

c

g-c

t-a

t-a

g-c

a-t

g-c

t-a g

t tttc a

a a !!!! t

t tttg aaag g

a !!!+ t a

t aaat a

a a g

t-aa

c-g

a-t

g-c

c t

t a

t g

gtg

mtRNA-His(gtg)

65 bases, %GC = 32.3

Sequence [10740,10804]

16.

g

t-a

a-t

t+g

t+g

c-g

c-g

a-t

a-t a

t tcgtt

ta a !!!!+ t

a ttcg agcag

a +!!! a t

a gagc g

tta a g

t-ag

a-t

g-c

c-g

a-t

c a

t g

gaa

mtRNA-Phe(gaa)

70 bases, %GC = 38.6

Sequence [12512,12581]

17.

c

c

g

t+g

c-g

c-g

t-a

a-t

a-t

a-t ga

t ccatt a

a g !!!!! t

g gtca ggtaa a

g :!!! c at

t aagt a

t g t

t-aa

t-a

a-t

g+t

a-t

c a

t a

ttt

mtRNA-(Lys|Asn)(ttt)

71 bases, %GC = 32.4

Sequence [14005,14075]

Overlap with 20: mtRNA-Ala(tgc) [14072,14139]

18.

a

g-c

g-c

c-g

c-g

t-a

g+t

g-c t

t ttcat g

aa a !!!!! t

g tttc aagta t

t !!!! t t

t aaag a

a a a

t-aa

t-a

t-a

g+t

a-t

t t

t a

tgc

mtRNA-Ala(tgc)

68 bases, %GC = 29.4

Sequence [14072,14139]

Overlap with 19: mtRNA-(Lys|Asn)(ttt) [14005,14075]

19.

t

a-t

a-t

t-a

g-c

t-a

a-t

a-t

a-t tg

a tttcc a

g a !+!!! a

a agcg agagg t

a !+!! t tg

a ttgc t

t a g

t-aa

g+t

c-g

g-c

g-c

t c

t g

tcg

mtRNA-Arg(tcg)

71 bases, %GC = 38.0

Sequence [14140,14210]

Overlap with 22: mtRNA-Asn(gtt) [14210,14278]

20.

g

t-a

a-t

a-t

a-t

t-a

g-c

a-t tt

a catt a

aa a !!!! t

a ccg gtaa a

g !!! t tt

a ggc t

agc a a

c-ggg

c-g

t-a

a-t

t.t

c a

t a

gtt

mtRNA-Asn(gtt)

69 bases, %GC = 31.9

Sequence [14210,14278]

Overlap with 21: mtRNA-Arg(tcg) [14140,14210]

21.

g

t-a

a-t

a-t

t-a

g-c

c-g

t-a

a-t g

c ctcta a

ag g !+!!! t

t gcc gggat t

g !!! t ta

a cgg a

a g t

a-taa

a-t

t-a

c-g

a-t

t t

t g

gat

mtRNA-Ile(gat)

70 bases, %GC = 37.1

Sequence [14279,14348]

22.

a

a-t

a-t

g-c

a-t

a-t

a-t

a-t

a-t tt

tca cctatt a

gt !!+!!! g

g gggtaa c

a t tt

gt t

aaa t

g-c

c-g

a-t

g+t

g+t

g-c

c a

t a

gct

D-loop mtRNA-Ser(gct)

70 bases, %GC = 34.3

Sequence [14702,14771]

tRNA Anticodon Frequency

AAA Phe GAA Phe 1 CAA Leu TAA Leu 1

AGA Ser GGA Ser CGA Ser TGA Ser 1

ACA Cys GCA Cys 1 CCA Trp TCA (Stop|Trp) 1

ATA Tyr GTA Tyr 1 CTA Pyl TTA Stop

AAG Leu GAG Leu CAG Leu TAG Leu 1

AGG Pro GGG Pro CGG Pro TGG Pro 1

ACG Arg GCG Arg CCG Arg TCG Arg 1

ATG His GTG His 1 CTG Gln TTG Gln 1

AAC Val GAC Val CAC Val TAC Val 1

AGC Ala GGC Ala CGC Ala TGC Ala 1

ACC Gly GCC Gly CCC Gly TCC Gly 1

ATC Asp GTC Asp 1 CTC Glu 1 TTC Glu 1

AAT Ile GAT Ile 1 CAT Met 1 TAT (Ile|Met) 1

AGT Thr GGT Thr CGT Thr TGT Thr 1

ACT Ser GCT Ser 1 CCT (Arg|Stop|Ser|Gly) TCT (Arg|Stop|Ser|Gly)

ATT Asn GTT Asn 1 CTT Lys TTT (Lys|Asn) 1

tRNA Codon Frequency

TTT Phe TTC Phe 1 TTG Leu TTA Leu 1

TCT Ser TCC Ser TCG Ser TCA Ser 1

TGT Cys TGC Cys 1 TGG Trp TGA (Stop|Trp) 1

TAT Tyr TAC Tyr 1 TAG Pyl TAA Stop

CTT Leu CTC Leu CTG Leu CTA Leu 1

CCT Pro CCC Pro CCG Pro CCA Pro 1

CGT Arg CGC Arg CGG Arg CGA Arg 1

CAT His CAC His 1 CAG Gln CAA Gln 1

GTT Val GTC Val GTG Val GTA Val 1

GCT Ala GCC Ala GCG Ala GCA Ala 1

GGT Gly GGC Gly GGG Gly GGA Gly 1

GAT Asp GAC Asp 1 GAG Glu 1 GAA Glu 1

ATT Ile ATC Ile 1 ATG Met 1 ATA (Ile|Met) 1

ACT Thr ACC Thr ACG Thr ACA Thr 1

AGT Ser AGC Ser 1 AGG (Arg|Stop|Ser|Gly) AGA (Arg|Stop|Ser|Gly)

AAT Asn AAC Asn 1 AAG Lys AAA (Lys|Asn) 1

Number of tRNA genes = 24

Number of D replacement loop tRNA genes = 1

tRNA GC range = 12.9% to 50.8%
